# High Molecular Weight Chitosan-Complexed RNA Nanoadjuvant for Effective Cancer Immunotherapy

**DOI:** 10.3390/pharmaceutics11120680

**Published:** 2019-12-14

**Authors:** Jin Joo Choi, Quoc-Viet Le, Dongho Kim, Young Bong Kim, Gayong Shim, Yu-Kyoung Oh

**Affiliations:** 1 College of Pharmacy and Research Institute of Pharmaceutical Sciences, Seoul National University, Seoul 08826, Korea; jinjoo.jc@snu.ac.kr (J.J.C.); lqviet.pharm@gmail.com (Q.-V.L.); 2 NA Vaccine Institute, Seoul 05854, Korea; dkim.gp@gmail.com; 3 Department of Biomedical Engineering, Konkuk University, Seoul 05029, Korea; kimera@konkuk.ac.kr

**Keywords:** RNA adjuvant, chitosan, nanoadjuvant, cancer vaccine, immunotherapy

## Abstract

Nucleic acid-based adjuvants have recently emerged as promising candidates for use in cancer vaccines to induce tumor-suppressing immune cells. In this study, we tested whether complexation of a nucleic acid-based adjuvant with chitosan (CTS) modulates immune adjuvant functions. As a nucleic acid-based adjuvant, we used toll-like receptor 3-recognizing RNA adjuvant (RA). Negatively charged RA formed nanoscale polyplexes with cationic CTS that possessed positive zeta potentials. RA/CTS polyplexes exerted dendritic cell (DC)-maturation effects without causing significant DC toxicity. This DC-maturation effect was CTS molecular weight dependent, with RA/CTS polyplexes with a CTS molecular weight of 340 kDa (RA/CTS 340K) producing the greatest effect. Subcutaneous injection of RA/CTS 340K polyplexes with the model tumor antigen ovalbumin exerted a preventive effect against challenge by ovalbumin-expressing tumor cells. It also provided greater inhibitory effects against a second challenge with the same tumor cells compared with other treatments. These protective effects of subcutaneous RA/CTS polyplex treatment were associated with the highest tumor antigen-specific humoral and cellular immune responses after tumor challenge, and with the greatest infiltration of CD4 helper T cell and CD8 T cell into the tumor tissues. Mice vaccinated with ovalbumin and RA/CTS polyplexes showed complete survival, even after repeated challenge with tumor cells. Our results suggest the potential of RA/CTS polyplexes as effective nanoadjuvants in the design of tumor vaccines and cancer immunotherapy.

## 1. Introduction

Cancer vaccines have been extensively studied for their potential to stimulate active antitumor immunity to fight against cancers [1]. For a cancer vaccine to be successful, it must elicit a robust and long-lasting immune response against tumor antigens. A key aspect in the formulation of cancer vaccines is inclusion of immunostimulatory agents, called vaccine adjuvants, which, together with tumor antigens, yield a stronger immune response. Alum derivatives are the vaccine adjuvants best known for their ability to provoke immunity and boost the humoral immune response [2]. However, alum derivatives alone have been reported to exert a limited cellular immune response, which is crucial for antitumor activity [2,3]. Thus, for the design of effective cancer vaccines, there is a need for new adjuvants that can boost cellular immune responses.

Because of their high biocompatibility and potency, nucleic acid-based adjuvants have emerged as a new class of adjuvants for cancer vaccines [4,5,6,7]. CpG and poly I:C are representative nucleic acid adjuvants that are capable of generating cytotoxic T lymphocytes, resulting in killing effects towards tumor cells [4,5,6]. Despite progress in this area, clinical trials of nucleic acid adjuvants for cancer vaccines have still shown insufficient preventive effects [7].

Nucleic acid adjuvants face some limitations for clinical use [7,8,9]. First, the intracellular targets of these nucleic acid adjuvants require that adjuvants efficiently penetrate into the cell [8]. Since nucleic acid adjuvants are negatively charged, it is difficult for them to permeate the negatively charged cell membrane. Secondly, nucleic acid adjuvants are extremely unstable in the presence of nucleases, such as DNase or RNase, found ubiquitously in body fluids. Rapid degradation after administration limits the efficacy of these adjuvants [7,9]. These attributes necessitate a delivery system that can protect nucleic acid adjuvants from enzymatic degradation and further enhance their uptake by dendritic cells (DCs).

Chitosan (CTS), a naturally derived polymer composed of linear polysaccharide, has been used intensively in biomedical engineering and drug delivery owing to its biodegradability and excellent biocompatibility [10]. CTS has also been adopted as a carrier for nucleic acid-based gene materials because of the abundance of versatile amine groups in the CTS backbone. The protonation of these amines at acidic pH causes CTS to become positively charged, enabling it to easily condense with genetic material to form nanoscale polyplexes. Through complexation, CTS is able to protect nucleic acids from enzyme-mediated degradation and improve the internalization of nucleic acid cargos into the cell. Although CTS is a potential carrier for nucleic acid adjuvant delivery, previous studies have shown that careful screening is required to identify polymers with molecular weights that are suited to the characteristics of nucleic acid materials [11,12].

**Figure 1:**
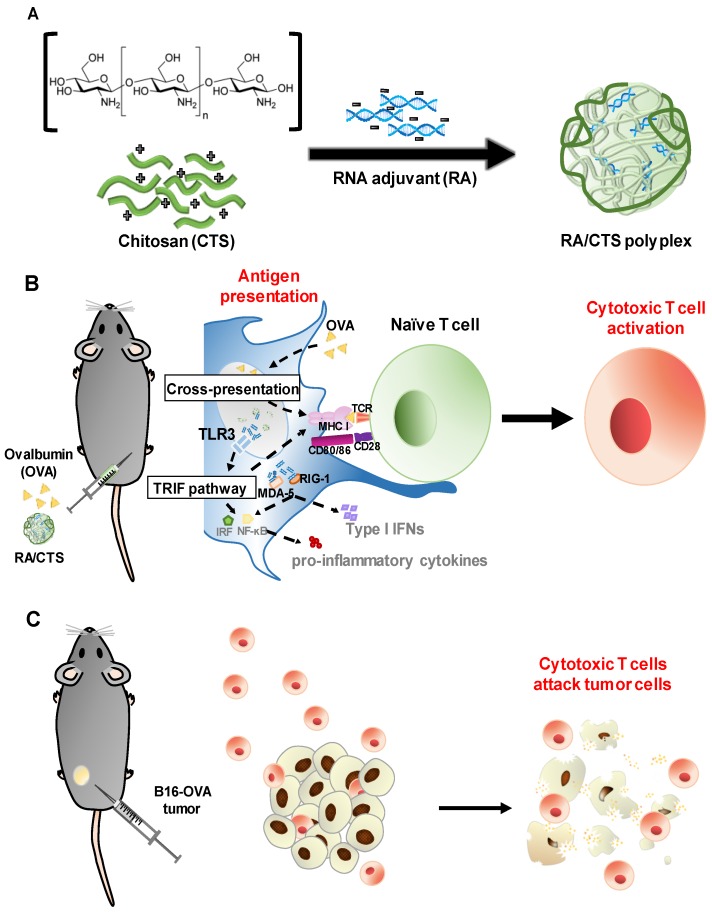
Schematic illustration of RA/CTS (RNA adjuvant/chitosan) polyplexes as an immune-modulating adjuvant for cancer immunotherapy. (**A**) RA was complexed with CTS through electrostatic interactions to form RA/CTS polyplexes. (**B**) Subcutaneous injection of RA/CTS together with ovalbumin (OVA) antigen activates dendritic cells (DCs), inducing their migration to nearby lymph nodes for subsequent antigen presentation. Naïve T cells presented with antigen epitopes via major histocompatibility complex I (MHC-I) molecules and co-stimulatory signals consequently differentiate into OVA-specific cytotoxic T cells. (**C**) These cytotoxic T cells are capable of killing OVA-expressing tumor cells, functioning as a preventive cancer vaccine.

Here, we tested CTS at various molecular weight ranges as a delivery system for nucleic acid adjuvants. In this study, we adopted poly I:C-derived double-stranded RNA adjuvant (RA) and ovalbumin (OVA) as a model nucleic acid adjuvant and antigen, respectively. RA/CTS polyplexes were formed by complexing negatively charged RA with cationic CTS polymer and then were tested in vivo for antitumor immune responses, as illustrated in Figure 1.

## 2. Materials and Methods

### 2.1. Preparation of RNA Adjuvant/CTS Polyplexes

RA/CTS polyplexes were formed by electrostatic interactions of anionic RA with cationic CTS with various molecular weight. The molecular weights of CTS (Sigma-Aldrich, St. Louis, MO, USA) are the following: 120K (50 to 190 kDa, catalog (cat.) # 448869); 141K (141 kDa, cat. # 42344), 250K (190 to 310 kDa, cat.# 448877), and 340K (310 to 375 kDa, cat.# 419419). Briefly, 10 mg of CTS was dissolved in 1% acetic acid at 400 μg/mL and stirred overnight at 50 °C. The CTS solution was then filtered through a 0.45-μm polycarbonate membrane filter (Millipore Corp., Billerica, MA, USA). Polyplexes were formed by physically mixing a CTS solution (pH 6.5) with RA (1 mg/mL in triple-distilled water) at various RA:CTS weight ratios, and incubating for 15 min. The resulting RA/CTS polyplexes formed at various ratios were stored at 4 °C until use.

### 2.2. Complexation and Stability of RA/CTS Polyplexes

Complexation of RA/CTS polyplexes was confirmed by gel retardation assay. RA/CTS polyplexes prepared with various molecular weights of CTS were incubated at room temperature for 15 min, then electrophoresed in 1% (*w*/*v*) agarose gels, and visualized using a Gel Doc XR+ Imaging System (Bio-Rad, Hercules, CA, USA).

### 2.3. Characterization of RA/CTS Polyplexes

RA/CTS polyplexes were characterized by morphology, size, and zeta potential. The morphology of RA/CTS polyplexes was examined by transmission electron microscopy (TEM) using a Talos L120C system (Thermo Fisher Scientific, Waltham, MA, USA). The size of CTS polyplexes was measured using dynamic light scattering technique, and zeta potential was measured by laser Doppler microelectrophoresis using an ELSZ-1000 instrument (Otsuka Electronics Co. Ltd., Osaka, Japan).

### 2.4. Animal Study Approval

All animal experiments followed Guidelines for the Care and Use of Laboratory Animals of the Institute of Laboratory Animal Resources. The research protocol was approved by the Institutional Review Committee for the Use of Animals at the College of Pharmacy, Seoul National University (approval #SNU- SNU-180914-4, 14 September, 2018).

### 2.5. Isolation of Bone Marrow-Derived Dendritic Cells (BMDCs)

BMDCs were isolated and differentiated from monocytes as previously described, with minor modifications [13]. Briefly, femurs and tibiae from 6-week-old C57BL/6 mice (Raon Bio, Yongin, Kyonggi-do, Korea) were isolated and flushed with complete RPMI media to collect bone marrow. Red blood cells were lysed to obtain monocyte pellets, which were suspended and cultured in Iscove’s modified Dulbecco’s medium supplemented with 10% fetal bovine serum (FBS; GenDEPOT, Barker, TX, USA), 100 units/mL penicillin, 100 mg/mL streptomycin (Gibco, Carlsbad, CA, USA), 20 ng/mL recombinant mouse granulocyte-macrophage colony-stimulating factor, 20 ng/mL recombinant mouse interlukin-4 (GenScript, Piscataway, NJ, USA), and 50 μM β-mercaptoethanol (Sigma-Aldrich). The medium was changed every 3 days, and BMDCs were harvested on day 7.

### 2.6. In Vitro Cell Viability Assay

In vitro cell viability was assessed by live-cell staining (Biomax Co., Seoul, Korea) and MTT (3-(4,5-dimethylthizol-2-yl)-2,5-diphenyltetrazolium bromide) assay (Sigma-Aldrich). BMDCs were seeded in 48-well plates and 96-well plates at approximately 95% confluence for live-cell imaging and MTT assay, respectively. After treating with various molecular weights of CTS (100 μg/mL) and RA/CTS polyplexes with an RA:CTS weight ratio of 1:10 for 24 h, the cells were stained with calcein (2 μM) for 15 min. Live cells were visualized by fluorescence microscopy (Leica DM IL, Wetzlar, Germany). For MTT assays, cells treated with formulations for 24 h were incubated with 250 μg/mL MTT for 2 h at 37 °C. After solubilization of formazans with dimethyl sulfoxide, the absorbance of each well was measured at 570 nm using a SpectraMax M5 plate reader (Molecular Devices, Sunnyvale, CA, USA).

### 2.7. In Vitro Assessment of DC Activation by RA/CTS Polyplexes

DC activation was evaluated by monitoring the expression of specific surface markers on splenic DCs by flow cytometry. Splenocytes were isolated from naïve C57BL/6 mice, seeded in 24-well plates, and treated with RA/CTS polyplexes for 24 h. The cells were then harvested and stained with FITC-conjugated anti-mouse CD11c antibody and APC-conjugated anti-mouse CD80 or CD86 (BioLegend, San Diego, CA, USA) antibodies for 1 h. After washing with 2% FBS in phosphate-buffered saline (PBS), the expression of these surface markers was analyzed using a BD FACSCalibur flow cytometer (BD Bioscience, San Jose, CA, USA) and Cell Quest Pro software. The data were also analyzed using FlowJo software v10.0.7 (BD Bioscience).

### 2.8. Intracellular Uptake Pathway Test

DC uptake pathways of RA/CTS polyplexes were explored using various chemicals inhibiting specific pathways of cellular entry. Genistein (200 μM; Sigma-Aldrich) inhibiting caveolae-mediated pathway [14], chlorpromazine (10 μM; Sigma-Aldrich) and sucrose (450 mM; Affymetrix, Cleveland, OH, USA) inhibiting clathrin-mediated pathway [15], and amiloride (1 mM; Sigma-Aldrich) inhibiting macropinocytosis [16] were used. Block-iT^TM^(BiT), a fluorescein-labelled dsRNA oligomer (Invitrogen, Carlsbad, CA, USA) complexed with CTS 340K (BiT/CTS) were used at a weight ratio of 1:20 to visualize the intracellular delivery. Briefly, BMDCs were seeded in 24-well plates, and treated with chemical inhibitors in RPMI media for 30 min prior to sample treatment. The BiT/CTS was treated to the BMDCs with a BiT concentration of 2 μg/mL. After 4 h treatment of samples, the cells were washed with 2% FBS in PBS and analyzed using BD FACSCalibur flow cytometer (BD Bioscience).

### 2.9. In Vivo Study of RA/CTS Polyplex-Mediated DC Activation

In vivo DC activation was evaluated by testing specific surface markers on splenocytes isolated from C57BL/6 mice. Mice were injected with RA/CTS polyplexes (CTS 340 kDa, 100 μg) at a RA:CTS weight ratio of 1:10, and splenocytes were isolated after 24 h. Isolated splenocytes were harvested and stained with FITC-conjugated anti-mouse CD11c antibody and APC-conjugated anti-mouse CD80 and CD86 (BioLegend). The expression of these surface markers on DCs was analyzed by gating on the CD11c(+) population.

### 2.10. In Vivo Study of Anticancer Efficacy

In vivo anticancer efficacy was tested after immunizing naïve C57BL/6 mice with 100 μg CTS or with RA/CTS polyplexes with a RA:CTS weight ratio of 1:10, and subsequently challenging with B16-OVA cells, a B16F10 tumor cell line stably transfected with OVA-encoding plasmid DNA (kindly provided by Prof. Youngro Byun, Seoul National University, Seoul, Korea). B16-OVA cells were cultured in Dulbecco’s Modified Eagle Medium (DMEM; Gibco) supplemented with 10% FBS (Gibco), 100 units/mL penicillin, and 100 mg/mL streptomycin (Gibco). RA/CTS polyplexes at a RA:CTS weight ratio of 1:10 (110 μg polyplex/mouse) and OVA (100 μg/mouse; Sigma-Aldrich) were injected subcutaneously into the right flank three times at 4-day intervals. Five days after the last treatment with RA/CTS polyplexes, B16-OVA cells (3 × 10^5^) were injected subcutaneously into the right flank. Tumors were measured with calipers, and volumes were calculated according to the equation, a × b^2^ × 0.5, where a is the longest and b is the shortest dimension [13]. Long-term protection against tumors afforded by vaccination was confirmed by re-challenging cured mice with B16-OVA cells, injected in the left flank. On day 69 after the first inoculation of B16-OVA cells, mice were re-challenged with 2 × 10^6^ B16-OVA cells, administered subcutaneously. Growth of tumors at re-challenged sites was monitored for 27 days. Naïve mice without vaccination were included as a control group.

### 2.11. Ex Vivo Analysis of Antigen-Specific Immune T Cell Responses

In vivo T cell priming induced by vaccination was evaluated by counting the number of interferon-γ (IFN-γ)-secreting cells in splenocytes using an ELISPOT assay (BD Bioscience). C57BL/6 mice were immunized with RA/CTS polyplexes and OVA antigen (100 μg) three times at 4-day intervals. Five days after the last immunization, mice were challenged with B16-OVA tumor cells. Two days after tumor challenge, splenocytes were isolated, seeded onto an IFN-γ ELISPOT plate (2 × 10^6^ cells/well) and re-stimulated with the class I (Kb)-restricted peptide epitope of ovalbumin, OVA_(257–264)_ (5 μg/mL; GenScript), for 24 h. IFN-γ–secreting cells, revealed by spot development, were counted manually using a stereomicroscope (SZ51; Olympus, Tokyo, Japan).

### 2.12. Measurement of OVA-Specific IgG Antibody Titer

An enzyme-linked immunosorbent assay (ELISA; Invitrogen, Carlsbad, CA, USA) was conducted to evaluate immunoglobulin (IgG) isotypes of anti-OVA antibodies in serum of immunized mice collected 6 and 10 days after tumor challenge. Briefly, 96-well ELISA plates were coated with 100 μg/mL OVA (Sigma-Aldrich) in PBS overnight at 4 °C. After washing with 0.05% Tween-20 in PBS (PBST), plates were blocked for 1 h in 10% FBS in PBS at 37 °C. After a thorough washing with PBST, diluted serum samples were added to each well and incubated with gentle shaking for 2 h. The plates were then washed again with PBST and incubated with horseradish peroxidase (HRP)-conjugated rat anti-mouse IgG (1 μg/mL in 10% FBS) for 2 h with gentle shaking. Finally, the plates were washed and 3,3′,5,5′-tetramethylbenzidine substrate was added. After 10 min, the reaction was stopped with sulfuric acid (0.16 M) and absorbance was detected at 450 nm with a SpectraMax M5 plate reader (Molecular Devices).

### 2.13. Assay of T Cell Infiltration

Tumor-infiltrating T cells were analyzed by flow cytometry. First, tumor tissues were excised on day 27 after tumor re-challenge and digested in collagenase (1 mg/mL in RPMI; Sigma-Aldrich) for 2 h at 37 °C with gentle stirring. Red blood cells were lysed and the resulting tumor cell suspension was passed through a 40-μm strainer. The cells were then stained with the following fluorescent antibodies: APC-conjugated anti-mouse CD3 antibody (BioLegend), PE-conjugated anti-mouse CD4 antibody (BioLegend), and PerCP/cyanine5.5-conjugated anti-mouse CD8a antibody (BioLegend). The percentage of CD3(+)CD4(+) and CD3(+)CD8(+) cells was analyzed using a BD FACSCalibur flow cytometer, and CellQuest Pro and FlowJo software v10.0.7 (BD Bioscience).

### 2.14. Measurement of Effector Memory T Cell Populations

Seven days after tumor re-challenge with B16-OVA tumor cells (2 × 10^6^ cells/mouse), inguinal lymph nodes closest to the tumor were isolated for analysis of effector memory T cell. Strainers (40 μm) were used to dissociate tissues into single cells. Red blood cells were lysed and the resulting cell populations were stained with the following fluorescent antibodies: FITC-conjugated anti-mouse CD3 antibody (BioLegend), PerCP/cyanine 5.5-conjugated anti-mouse CD8a (BioLegend), PE-conjugated anti-mouse CD62L (BioLegend), and APC-conjugated anti-mouse CD44 (BioLegend). The percentage of effector memory T cells [CD3(+)CD8(+)CD44^high^CD62^low^] was analyzed using a BD FACSCalibur flow cytometer (BD Bioscience), CellQuest Pro and FlowJo software.

### 2.15. Statistics

Data were analyzed by one-way analysis of variance (ANOVA), followed by Student–Newman–Keuls post hoc test. SigmaStat software (version 12.0; Systat Software, Richmond, CA, USA) was used for all statistical analyses, and *p*-values less than 0.05 were considered statistically significant.

## 3. Results

### 3.1. Characterization of RA/CTS Polyplexes

**Figure 2:**
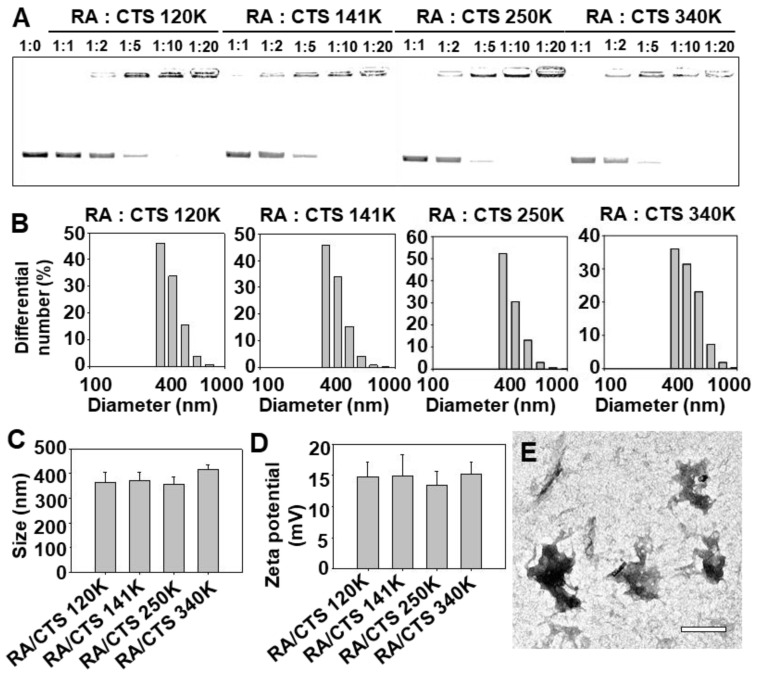
Characterization of RA/CTS polyplexes. (**A**) Polyplex formation between RA and CTS at various weight ratios was tested by gel retardation assay on agarose gels. Size distribution (**B**) and mean size (**C**) of RA/CTS polyplexes formulated with CTS at various molecular weights were measured by dynamic light scattering. (**D**) Zeta potentials were obtained from RA/CTS polyplexes formed at various CTS molecular weights. (**E**) Morphology of RA/CTS polyplexes using CTS 340 kDa (1:10, *w*/*w*), observed by TEM imaging. Scale bar, 500 nm.

The formation and physicochemical properties of RA/CTS polyplexes were characterized by gel retardation, and measurement of size, zeta potential, and morphology. Gel retardation studies revealed that RA/CTS polyplexes formed at RA:CTS weight ratios of 1:10 and 1:20 showed no migration of uncomplexed free RA, regardless of the molecular weight of CTS (Figure 2A). On the basis of these results, we used RA/CTS polyplexes at a 1:10 ratio throughout the study. Similar patterns of size distributions were observed for various CTS-complexed RAs (Figure 2B). There were no significant differences between groups in terms of average size (Figure 2C) and zeta potentials (Figure 2D) of RA/CTS with various molecular weights of CTS. TEM imaging showed the formation of nanoscale RA/CTS polyplexes (Figure 2E).

### 3.2. Cytotoxicity of RA/CTS Polyplexes

**Figure 3:**
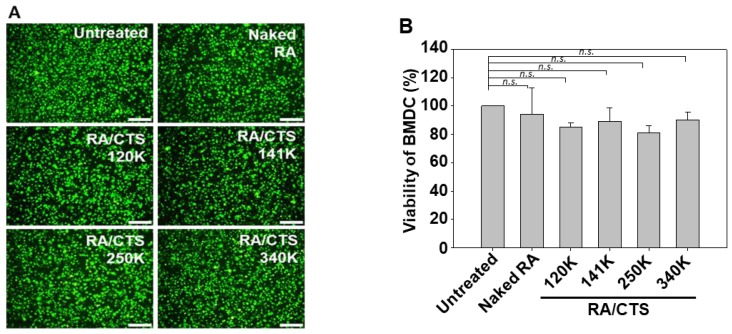
Viability of BMDCs treated with RA/CTS polyplexes. Bone marrow-derived dendritic cells (BMDCs) were treated for 24 h with RA/CTS polyplexes formed with various molecular weights of CTS. The viability of BMDCs was examined using live-cell imaging (**A**) and MTT assay (**B**). Size bar: 50 μm. n.s.: not significantly different.

RA/CTS polyplexes did not exert significant toxicity against BMDCs. Live-cell staining (Figure 3A) showed that the density of live cells was not notably decreased upon treatment with RA/CTS polyplexes formed with various molecular weights of CTS. Consistent with these live-cell staining data, MTT assays revealed that the viability of BMDCs did not significantly change, regardless of RA/CTS polyplex treatment (Figure 3B).

### 3.3. In Vitro DC Maturation-Inducing Effect of RA/CTS Polyplexes

**Figure 4:**
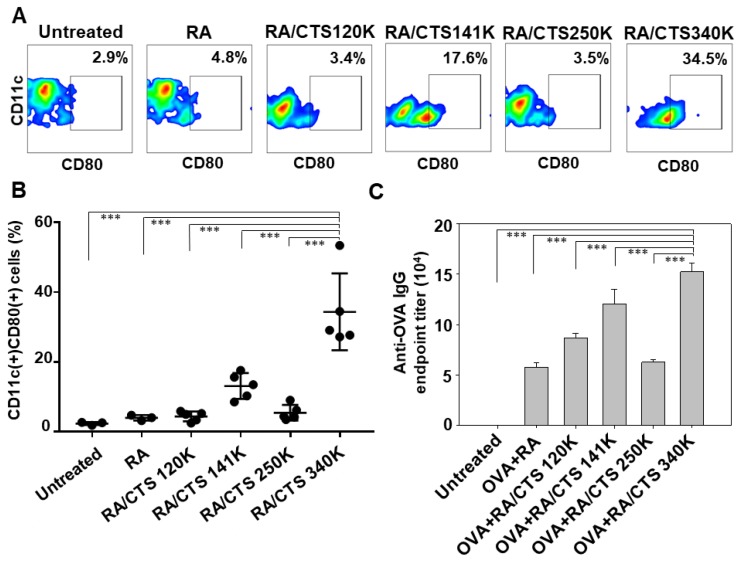
Immune-boosting effect of various RA/CTS polyplexes. Splenic DCs were treated with various formulations of RA/CTS polyplexes. After 24 h, the surface expression of CD80 on DCs were measured (**A**) and quantified (**B**) by flow cytometry (*** *p* < 0.005). (**C**) Naïve C57BL/6 mice were vaccinated 3 times at 4-day intervals with RA/CTS physically mixed with OVA antigen (100 μg). Five days after the last vaccination (day 0), mice were inoculated subcutaneously with B16-OVA cells. Blood samples were collected 6 days after inoculation for serum anti-OVA IgG titration at different dilution factors. (Data shown as mean ± SEM, *n* = 4). *** *p* < 0.005.

RA/CTS polyplexes exerted molecular weight-dependent DC maturation effects. The maturation of splenic DCs was evaluated by monitoring the surface expression of CD80. The population of CD11c(+)CD80(+) cells was the highest after treatment with RA/CTS polyplexes with a CTS molecular weight of 340K (Figure 4A,B). Specifically, the population of CD11c(+)CD80(+) cells following treatment with RA/CTS 340K polyplexes was 7.2-fold higher than that following treatment with naked RA (4.8% ± 0.8%) and 14.4-fold higher compared with untreated cells (Figure 4B). To assess the molecular weight dependence of immune responses, we examined immune responses after vaccination with RA/CTS polyplexes, followed by tumor challenge (Figure 4C). Serum anti-OVA IgG antibody levels were used as indicators immune response against OVA antigen, which is tumor specific antigen of B16-OVA tumor model. Of the molecular weights tested, RA/CTS 340K induced the highest endpoint titer of serum anti-OVA IgG antibodies compared to other CTS types or RA only treatment (Figure 4C). Based on these results, RA/CTS 340K polyplexes were chosen as the formulation for in vivo studies.

### 3.4. In Vivo DC Maturation by RA/CTS Polyplexes

**Figure 5:**
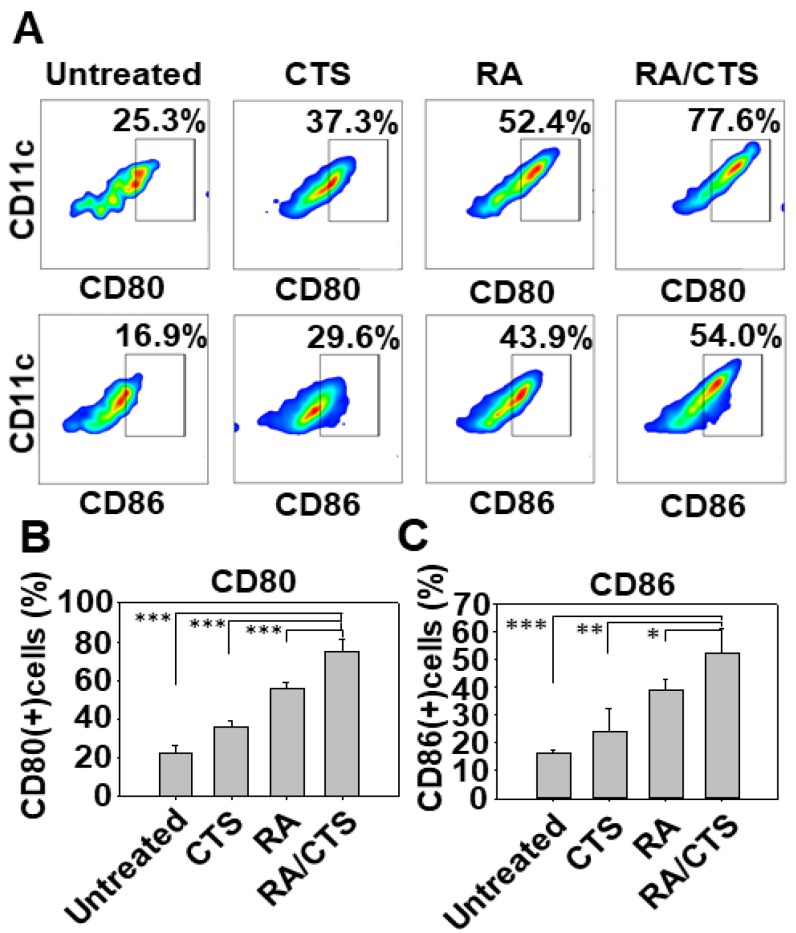
In vivo DC maturation effect of RA/CTS 340K polyplexes. Naïve C57BL/6 mice were administered RA/CTS 340K polyplexes at an RA dose of 10 μg. After 24 h, splenocytes from these mice were isolated and the surface expression of CD80 and CD86 among CD11c(+) cells was measured by flow cytometry (**A**). CD80(+) (**B**) and CD86(+) (**C**) DC populations were quantified for each group. Results are expressed as mean ± SEM (*n* = 3). * *p* < 0.05; ** *p* < 0.01; *** *p* < 0.005.

Treatment with RA/CTS 340K polyplexes enhanced the in vivo maturation of DCs, determined using CD80 and CD86 as DC maturation markers. The RA/CTS 340K-treated group showed the highest population of CD80(+) and CD86(+) cells compared with other groups (Figure 5A). Specifically, the percentage of CD80(+) cells in this group was 74.8% ± 6.6% (Figure 5B), and the percentage of CD86(+) cells was 52.2% ± 8.8% (Figure 5C).

### 3.5. In Vivo Tumor Growth-Inhibition Effect

**Figure 6:**
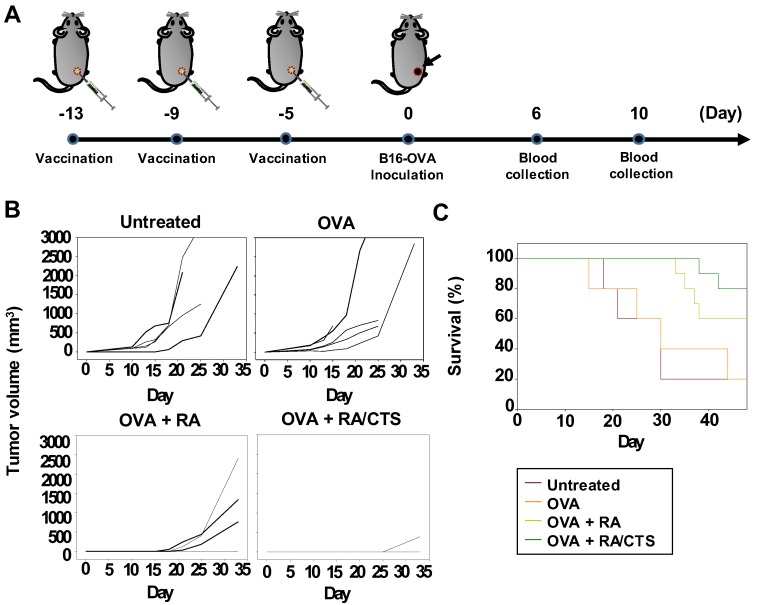
In vivo antitumor effects of RA/CTS 340K polyplexes. (**A**) Timeline for in vivo experiments. Naïve C57BL/6 mice were vaccinated three times at 4-day intervals (days -13, -9, -5) with a mixture of RA/CTS 340K polyplexes plus OVA antigen (100 μg). Five days after the last vaccination (day 0), mice were inoculated subcutaneously with B16-OVA cells (3 × 10^5^). (**B**) Tumor volumes were measured until day 33 after tumor inoculation. (**C**) Survival of mice was monitored until day 48. *n* = 5 (untreated, OVA) and *n* = 10 (OVA + RA, OVA + RA/CTS).

Treatment with RA/CTS 340K polyplexes enhanced the antitumor effects of OVA against B16-OVA tumors. Mouse vaccination schedules and challenge with B16-OVA tumors are illustrated in Figure 6A. Three vaccinations of OVA alone did not prevent the growth of B16-OVA tumors (Figure 6B). Although vaccination with OVA and RA mixture modestly attenuated tumor growth (Figure 6B) and showed some protection compared with OVA alone (Figure 6C), it was far less effective than vaccination with OVA and RA/CTS 340K polyplexes, which almost completely abrogated B16-OVA tumor growth (Figure 6B) and dramatically enhanced mouse survival (Figure 6C).

### 3.6. In Vivo Immune Adjuvant Effects of RA/CTS Polyplexes

**Figure 7:**
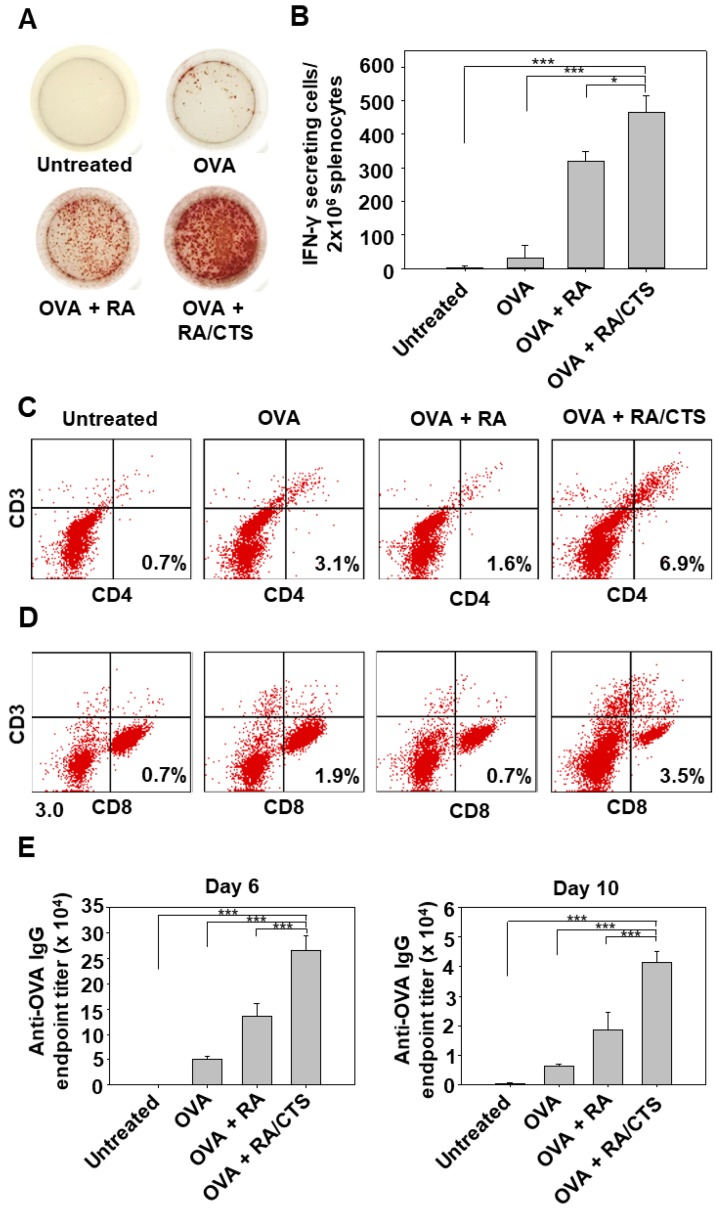
Post-vaccination humoral and cellular immune responses. Naïve mice were vaccinated three times with OVA in various formulations at 4-day intervals. Five days after the last vaccination, mice were inoculated with B16-OVA tumor cells (3 × 10^5^). Splenocytes were isolated 2 days after tumor challenge for ex vivo IFN-γ ELISPOT (**A**). IFN-γ–secreting cells were counted using a stereomicroscope (*n* = 4); * *p* < 0.05; *** *p* < 0.005) (**B**). Tumor-infiltrating T cells in tumors were analyzed for CD3(+)CD4(+) T helper cells (**C**) and CD3(+)CD8(+) cytotoxic T cells (**D**) by flow cytometry on day 27. Blood samples were collected 6 and 10 days after inoculation for serum anti-OVA IgG titration. Anti-OVA IgG levels were measured by ELISA in serum samples diluted at different ratios (*n* = 5) (*** *p* < 0.005) (**E**).

To explore mechanisms underlying the tumor preventive effects of RA/CTS polyplexes, we examined cellular and humoral immune responses after tumor challenge. The cellular immune response was evaluated by assessing the functionality of splenocytes isolated from immunized mice 2 days after tumor challenge. Mice treated with a mixture of OVA and RA/CTS 340K polyplexes showed a greater number of IFN-γ–secreting cells compared with other groups (Figure 7A). Specifically, the number of IFN-γ–secreting cell in the RA/CTS polyplex group was 14.5- and 1.5-fold higher than that in groups treated with OVA alone or OVA plus RA, respectively (Figure 7B). Treatment with RA/CTS polyplexes also increased the population of tumor tissue-infiltrating T cells, producing the highest percentage of CD4(+) (Figure 7C) and CD8(+) (Figure 7D) T cells. In addition to cellular immune responses, humoral immune responses were increased by treatment with RA/CTS 340K polyplexes. Vaccination of mice with the mixture of OVA plus RA/CTS polyplexes induced the highest levels of anti-OVA IgG antibodies in serum at days 6 and 10 after tumor inoculation. At day 6, the anti-OVA IgG titer of the group treated with OVA plus RA/CTS was 1.9- and 5.2-fold higher than the groups treated with OVA+RA, and OVA alone, respectively (Figure 7E).

### 3.7. Antitumor Effect Against Secondarily Challenged Tumor

**Figure 8:**
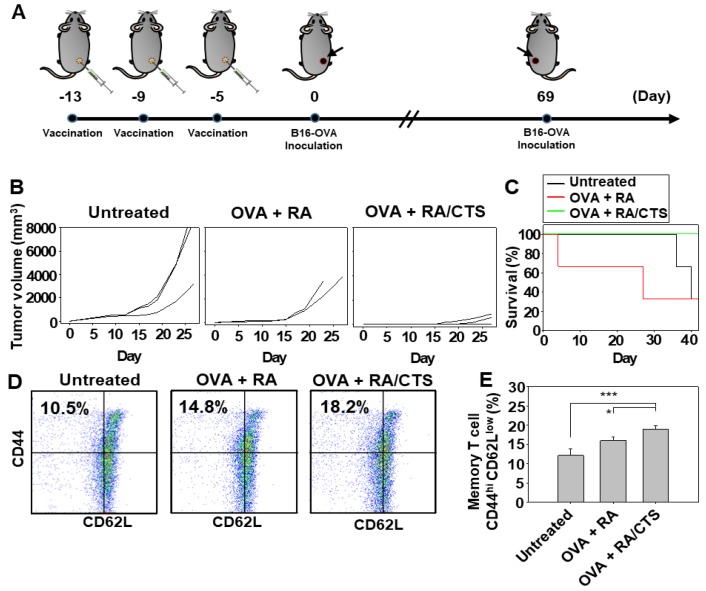
Long-term protective effect of RA/CTS polyplexes against tumor recurrence. Naïve mice were vaccinated three times with RA/CTS physically mixed with OVA antigen at 4-day intervals (**A**). Five days after the last vaccination, mice were inoculated with B16-OVA cells (3 × 10^5^). At 69 days after inoculation, mice were re-inoculated with B16-OVA cells (2 × 10^6^) and tumor size was measured for 27 days (*n* = 3) (**B**). Survival rates were monitored for 42 days from tumor re-challenge (**C**). Nine days after tumor re-challenge, tumor-draining lymph nodes were collected for analysis of effector memory T cells by flow cytometry (**D**). The percentage of effector memory T cells, CD3(+)CD8(+)CD44^high^Cd62L^low^, in each group (*n* = 3) was quantified (* *p* < 0.05; *** *p* < 0.005) (**E**).

In addition to protecting against the growth of primary tumors, the mixture of OVA with RA/CTS polyplexes also protected against secondary tumor challenge. The schedule of first and second tumor challenges is illustrated in Figure 8A. Compared to the group vaccinated three times with the mixture of OVA and free RA, the group treated with OVA plus RA/CTS polyplexes showed significantly decreased growth of secondarily challenged B16-OVA cell tumors (Figure 8B). On day 40 after the second challenge, mouse survival was 100% in the group vaccinated with the mixture of OVA and RA/CTS polyplex, but only 30% in the group vaccinated with the mixture of OVA and free RA (Figure 8C).

To investigate the mechanism of the antitumor effect against secondarily challenged tumors, we quantified effector memory T cells. The group treated with the mixture of OVA and RA/CTS polyplexes induced significantly higher populations of effector memory T cells [CD3(+) CD8(+) CD44^high^ CD62L^low^] in inguinal lymph nodes compared with other groups (Figure 8D,E).

## 4. Discussion

In this study, we demonstrated that RA/CTS polyplexes could function as an effective adjuvant for cancer vaccines. RA/CTS polyplexes significantly induced humoral and cellular immune responses against a model tumor antigen. The use of RA/CTS polyplexes together with a model tumor antigen exerted long-term prevention of secondarily challenged tumor cells expressing the model antigen.

RA could be condensed to nanoscale complexes with CTS of various molecular weights. The driving force for the formation of RA/CTS polyplexes is the electrostatic interaction between negatively charged RA and positively charged CTS. The cationic surface charge of particle provided stronger interaction with negatively charged cell membrane, inducing more effective cellular uptake by antigen presenting cells. Therefore the nanoscale sizes of positively charged RA/CTS polyplexes would increase phagocytosis as compared with uncondensed, free RA [17]. The main cellular uptake pathways of RA/CTS polyplexes would be clathrin-mediated endocytosis and macropinocytosis (Supplementary Figure S1). We observed that the cellular uptake of RA/CTS was significantly decreased upon the inhibition of clathrin-mediated endocytosis by chlorpromazine/sucrose, and macropinocytosis by amiloride. Similar to our observation, it has been reported that CTS-based nanoparticles were internalized to the cells by clathrin-mediated pathway and macropinocytosis [18]. At the optimized RA:CTS complexation ratio of 1:10, the surface of polyplexes had a net positive charge. The charge of OVA protein, used as a model tumor antigen, is known to be 1 to −27 mV [19,20]. The positive charge of RA/CTS polyplexes may support the interaction between the polyplex and tumor antigen. Because most human antigens possess a negative charge [21,22], RA/CTS polyplexes could potentially interact with other tumor antigen proteins in future applications.

The immunostimulatory efficacy of RA/CTS polyplexes was found to be affected by the molecular weight of CTS, with a molecular weight of 340K producing the greatest immunostimulatory effect. We observed that the immune-boosting effect did not gradually increase with the molecular weight of CTS. RA/CTS (140 K) polyplexes showed higher DC maturation (Figure 4A,B) and humoral immune responses than RA/CTS (250 K) polyplexes (Figure 4C). This result is thought to be attributed in part to the wide ranges of CTS sizes used in this study. CTS 120K, 250K, and 340K are the average sizes, and categorized as low (50 to 190 kDa), medium (190 to 310 kDa) and high (310 to 375 kDa) molecular weight, respectively. Unlike these CTS with a wide range of molecular weights, CTS 141 K was supplied as homogenous size. The exact mechanisms need to be studied further. However, we cannot exclude the possibility that the homogeneity might play a role in boosting immune responses. To test the feasibility, the comparison of homogenous sizes of CTS needs to be done in the future. Although additional studies will be required to determine why 340K CTS was most effective, it has been reported that CTS with higher molecular weights possesses a greater ability to intertwine with nucleic acids because of its highly positive charge [23]. Thus, the stronger effect of 340K CTS might be attributable to the stronger interactions between RA and CTS. It has also been reported that the transfection efficiency of plasmid DNA is dependent on the molecular weight of CTS [24].

In addition to the contribution of the physicochemical features of CTS, it is possible that higher molecular weight CTS could have higher immunostimulatory effects per se. Several studies have reported that antimicrobial and antioxidant effects of CTS are affected by its molecular weight [25,26,27]. The DC maturation-inducing effect of CTS was reported to occur through stimulation of the STING pathway [28]. In this latter study, CTS with a broad molecular weight (150–400 kDa) was used. At this point, the immunostimulatory effect of high molecular weight CTS itself is poorly understood. Thus, we cannot exclude the possibility that unique molecular and cellular actions of high molecular weight CTS may have contributed to the enhanced DC maturation effect.

RA, a defined molecular weight-derivative of poly I:C, behaves as a toll-like receptor (TLR) 3 agonist. Poly I:C, a synthetic double-stranded RNA, is also known to act as an agonist of TLR3. The TLR3-mediated, toll/interleukin-1 receptor domain-containing, adaptor-inducing interferon-B pathway leads to production of pro-inflammatory cytokines (e.g., interleukin-6) and chemokines, and most importantly, causes maturation of DCs [29]. Although poly I:C is a very effective adjuvant, the clinical translation of poly I:C for human use has remained limited owing to issues related to its undefined structure and heterogeneity [30]. Indeed, poly I:C activity was reported to be molecular weight-dependent, and varied depending on the commercial source [31]. RA/CTS polyplexes may overcome the molecular weight variation issue of poly I:C for human use.

In this study, we used the subcutaneous route for vaccination of mice with OVA and RA/CTS polyplexes. Systemic administration of nucleic acid adjuvants may induce a massive cytokine storm that results in systemic side effects [5]. Therefore, local injection such as intradermal or subcutaneous routes may be the preferred strategy for RA/CTS polyplex adjuvants. Moreover, intramuscular or subcutaneous routes are the most clinically acceptable for vaccination because of their relatively easy access, potential for consistent delivery, and sufficient immunogenicity [32]. The subcutaneous route is known to provide efficient lymphatic drainage, which could increase vaccine delivery to nearby draining lymph nodes [33,34].

The primary goal of cancer vaccination is to prime tumor antigen-specific T cells to recognize and eliminate tumor cells. RA, through activation of TLR3 signaling, causes DC maturation and enhances cross-presentation of antigens through upregulation of MHC-I and CD80/86 expression on DCs [17]. This enhanced antigen presentation activates naïve CD8(+) T cells as well as CD4(+) T cells. Generation of CD8(+) cytotoxic T cells is critical for antitumor activity, and DCs are among the most potent cross-presenting APCs in vivo [4]. RA/CTS polyplexes are thought to enhance cellular and humoral immune responses [35,36], possibly through DC maturation [28]. The induction of IFN-γ–producing T cells in the RA/CTS polyplex-treated group supports the conclusion that RA/CTS polyplexes can cause cross-antigen presentation and cytotoxic T cell priming.

In this study, B16-OVA cells were chosen as a tumor model for evaluation of RA/CTS complexes as a prophylactic cancer vaccine. Melanoma is known to be an aggressive cancer with a high risk of recurrence and metastasis. Our studies of the protective efficacy of vaccination in mice challenged twice with B16-OVA tumor cells showed that suppression of challenged tumor growth was attributable to the increased infiltration of both CD4(+) and CD8(+) T lymphocytes into the tumor tissues. The long-term tumor-preventive effect observed in OVA and RA/CTS polyplex-vaccinated mice could be explained by the increase in effector memory T cells [13,37].

## 5. Conclusions

In this study, we provided evidence that RA/CTS polyplexes could serve as an effective nanoadjuvant of tumor antigens. Through complexation with CTS, the TLR3-type adjuvant effect of RA was significantly enhanced. Although we used OVA as a model tumor antigen, RA/CTS polyplexes could be combined with other tumor antigens to induce immune responses that prevent the uncontrolled growth of tumors expressing different specific antigens.

## Supplementary Materials

The following are available online at https://www.mdpi.com/1999-4923/11/12/680/s1, Figure S1: Effects of endocytic inhibitors on the uptake of block-iT/CTS 340K (BiT/CTS) polyplexes by BMDCs.
