# Supplementary material for: High Molecular Weight Chitosan-Complexed RNA Nanoadjuvant for Effective Cancer Immunotherapy
Source: Pharmaceutics. 2019 Dec 14;11(12):680. doi: 10.3390/pharmaceutics11120680 (PMC6969902; doi:10.3390/pharmaceutics11120680)
Supplement: Supplementary file 1 [file pharmaceutics-11-00680-s001.pdf]

pharmaceutics

Supplementary Materials: High Molecular Weight Chitosan-Complexed RNA Nanoadjuvant for Effective Cancer Immunotherapy

Jin Joo Choi, Quoc-Viet Le, Dongho Kim, Young Bong Kim, Gayong Shim and Yu-Kyoung Oh

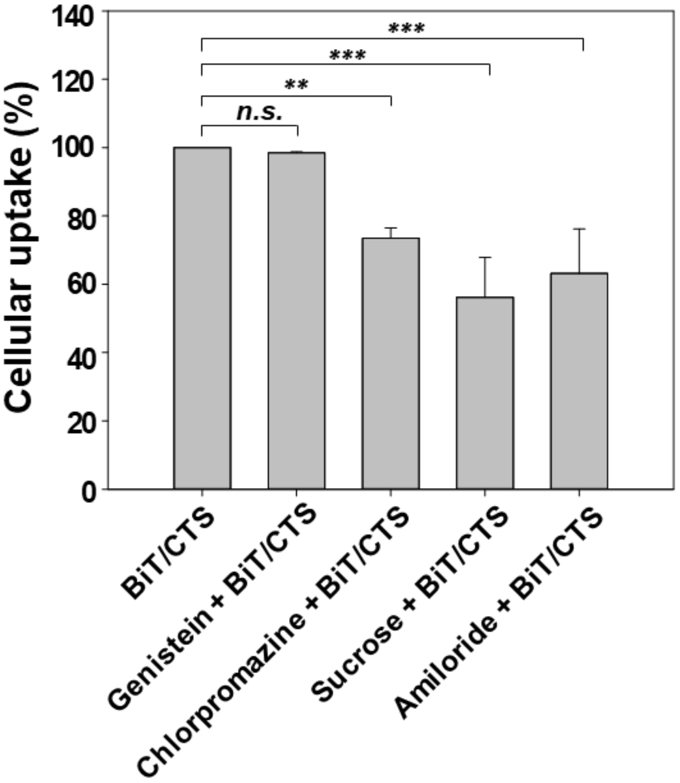

Figure S1. Effects of endocytic inhibitors on the uptake of block-iT/CTS 340K (BiT/CTS) polyplexes by BMDCs. After pre-treatment of genistein (200μM), chlorpromazine (10μM), sucrose (450mM), amiloride (1mM) in media for 30 minutes, BiT/CTS polyplexes were then added for 4 hours. Then the cells were washed for flow cytometry. Data shown as mean ± SEM, n = 3.
